# Author Correction: PARP14 and PARP9/DTX3L regulate interferon-induced ADP-ribosylation

**DOI:** 10.1038/s44318-024-00247-6

**Published:** 2024-09-25

**Authors:** Pulak Kar, Chatrin Chatrin, Nina Đukić, Osamu Suyari, Marion Schuller, Kang Zhu, Evgeniia Prokhorova, Nicolas Bigot, Domagoj Baretić, Juraj Ahel, Jonas Damgaard Elsborg, Michael L Nielsen, Tim Clausen, Sébastien Huet, Mario Niepel, Sumana Sanyal, Dragana Ahel, Rebecca Smith, Ivan Ahel

**Affiliations:** 1 Sir William Dunn School of Pathology, University of Oxford, Oxford, OX1 3RE UK; 2 Department of Biological Sciences, SRM University-AP, Amaravati, 522502 India; 3 Univ Rennes, CNRS, IGDR (Institut de génétique et développement de Rennes) - UMR 6290, BIOSIT – UMS3480, F-35000 Rennes, France; 4 Research Institute of Molecular Pathology (IMP), Vienna BioCenter, Vienna, Austria; 5 Proteomics Program, Novo Nordisk Foundation Center for Protein Research, Faculty of Health and Medical Sciences, University of Copenhagen, Blegdamsvej 3B, 2200 Copenhagen, Denmark; 6 Medical University of Vienna, Vienna, Austria; 7 Ribon Therapeutics, Cambridge, MA 02140 USA

## Abstract

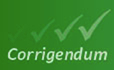

**Correction to:** *The EMBO Journal* (2024) 43: 2929–2953. 10.1038/s44318-024-00126-0 | Published online 4 June 2024

**The author list is corrected**.

Domagoj Baretić was omitted from the author list.

Domagoj Baretić is added to the paper as the 9th author, with the affiliation: ^1^Sir William Dunn School of Pathology, University of Oxford, Oxford OX1 3RE, UK.

All authors agree to this correction.

The original article has been corrected.

