# Supplementary material for: PARP14 and PARP9/DTX3L regulate interferon-induced ADP-ribosylation
Source: EMBO J. 2024 Jun 4;43(14):7. doi: 10.1038/s44318-024-00126-0 (PMC11251020; doi:10.1038/s44318-024-00126-0)
Supplement: Supplementary file 1 — Appendix [file 44318_2024_126_MOESM1_ESM.pdf]

PARP14 and PARP9/DTX3L regulate interferon-induced ADP-ribosylation

Pulak Kar[1,2]†, Chatrin Chatrin[1,]†, Nina Đukic[1]†, Osamu Suyari[1], Marion Schuller[1], Kang Zhu[1], Evgeniia Prokhorova[1], Nicolas Bigot[3], Juraj Ahel[4], Jonas Damgaard Elsborg[5], Michael L Nielsen[5], Tim Clausen[4,6], Sébastien Huet[3], Mario Niepel[7], Sumana Sanyal[1], Dragana Ahel[1], Rebecca Smith[1,*], Ivan Ahel[1,8,*]

[1] Sir William Dunn School of Pathology, University of Oxford, Oxford OX1 3RE, UK
[2] Department of Biological Sciences, SRM University-AP, Amaravati 522502, India
[3] Univ Rennes, CNRS, IGDR (Institut de génétique et développement de Rennes) - UMR 6290, BIOSIT – UMS3480, F- 35000 Rennes, France
[4] Research Institute of Molecular Pathology (IMP), Vienna BioCenter, Vienna, Austria.
[5] Proteomics program, Novo Nordisk Foundation Center for Protein Research, Faculty of Health and Medical Sciences, University of Copenhagen, Blegdamsvej 3B, 2200, Copenhagen, Denmark
[6] Medical University of Vienna, Vienna, Austria
[7] Ribon Therapeutics, Cambridge, MA 02140, USA
[8] Lead Contact
† Contributed Equally
* Correspondence to : R.S (rebecca.smith@path.ox.ac.uk) and I.A (ivan.ahel@path.ox.ac.uk)

This PDF contains:
Appendix Table S1: sgRNA used for knockout generation
Appendix Table S2: Antibodies used in this study
Appendix Table S3: qPCR primers used in this study

Appendix Table S1: sgRNA used for knockout generation

Guide	Sequence 5'-3'
PARP14 002	CGGCGAGTGTGAGGTCCGCC
PARP14 001	CTCCCGACCTCTTCGGGCTC
PARP14 003	CGGCGAGTGTGAGGTCCGCC
DTX3L 1	CACCACAAAACATCCCGGTTTGG
DTX3L 2	AATACGGCAATTTCTGGATGTGG

Appendix Table S2: Antibodies used in this study

Target	Host	Company	Reference	Dilution in WB	Dilution in IF	Dilution in IP
PARP14	Rabbit	Abcam	Ab229756	1:1000	-	-
PARP9	Rabbit	Millipore	AB10618	1:1000	1:500	-
DTX3L	Mouse	Santa Cruz	Sc-1000627	1:1000	-	-
STAT1	Rabbit	Cell Signaling	9172	1:3000	-	-
pSTAT1 (Tyr701)	Rabbit	Cell Signaling	7649	1:3000	-	-
GAPDH	Mouse	Merck	MAB374	1:3000	-	-
mono-ADPr (HRP) AbD43647 (HRP coupled)	-		Longarini et al 2023	1:3000/1:5000	-	-
mono-ADPr AbD43647 (IgG coupled)	Mouse		Longarini et al 2023	-	1:1000	-
STAT6	Rabbit	Cell Signaling	9362	1:2000	-	-
Histone H3	Rabbit	Sigma-Aldrich	06-755	1:4000	-	-
FLAG	Rabbit	Sigma-Aldrich	F7425	1:3000	-	-
poly/mono ADPr	Rabbit	Cell Signaling	83732	1:1000	1:500	-
PARP14	Rabbit	This study	N/A	-	15 µg/mL	1:100
PARP14	Rabbit	Abcam	ab224352	-	1:100	-
GFP	Rabbit	Abcam	Ab290	1:3000	-	-
GFP	Mouse	Rockland	600-301-215	-	1:100	-
beta Tubulin	Rabbit	Abcam	ab6046	1:3000	-	-
Ubiquitin	Mouse	Santa Cruz	Sc-8017		1:100	-
Ubiquitin	Rabbit	Abcam	Ab134953	-	1:100	-
Anti-mouse Alexa Fluor 488	Goat	Thermo Fisher Scientific	A28175	-	1:500	-
Anti-rabbit Alexa Fluor 647	Donkey	Thermo Fisher Scientific	A32795	-	1:500	-
HRP-conjugated anti-mouse	Goat	Agilent	P0447	1:3000	-	-
HRP-conjugated anti-rabbit	Swine	Agilent	P0399	1:3000	-	-
IgG Isotype Control	Rabbit	Invitrogen	02-6102	-	-	1:300

Appendix Table S3: qPCR primers used in this study

Gene	Forward (5'-3')	Reverse (5'-3')
HPRT1	GCGTCGTGATTAGCGATGATG	CTCGAGCAAGTCTTTCAGTCC
GAPDH	AAATCAAGTGGGGCGATGCTG	GCAGAGATGATGACCCTTTTG
PARP14	CTGCAGTCCGGCGGAGAG	TTTGGTCTTGGATTCTTTTGTTAGAAGTTC
PARP12	TCAAGAATAAGAGCTCTGCCCC	TGATCACCCTCCTCCTGGCT
