# Supplementary figures and images for: PARP14 and PARP9/DTX3L regulate interferon-induced ADP-ribosylation
Source: EMBO J. 2024 Jun 4;43(14):7. doi: 10.1038/s44318-024-00126-0 (PMC11251020; doi:10.1038/s44318-024-00126-0)

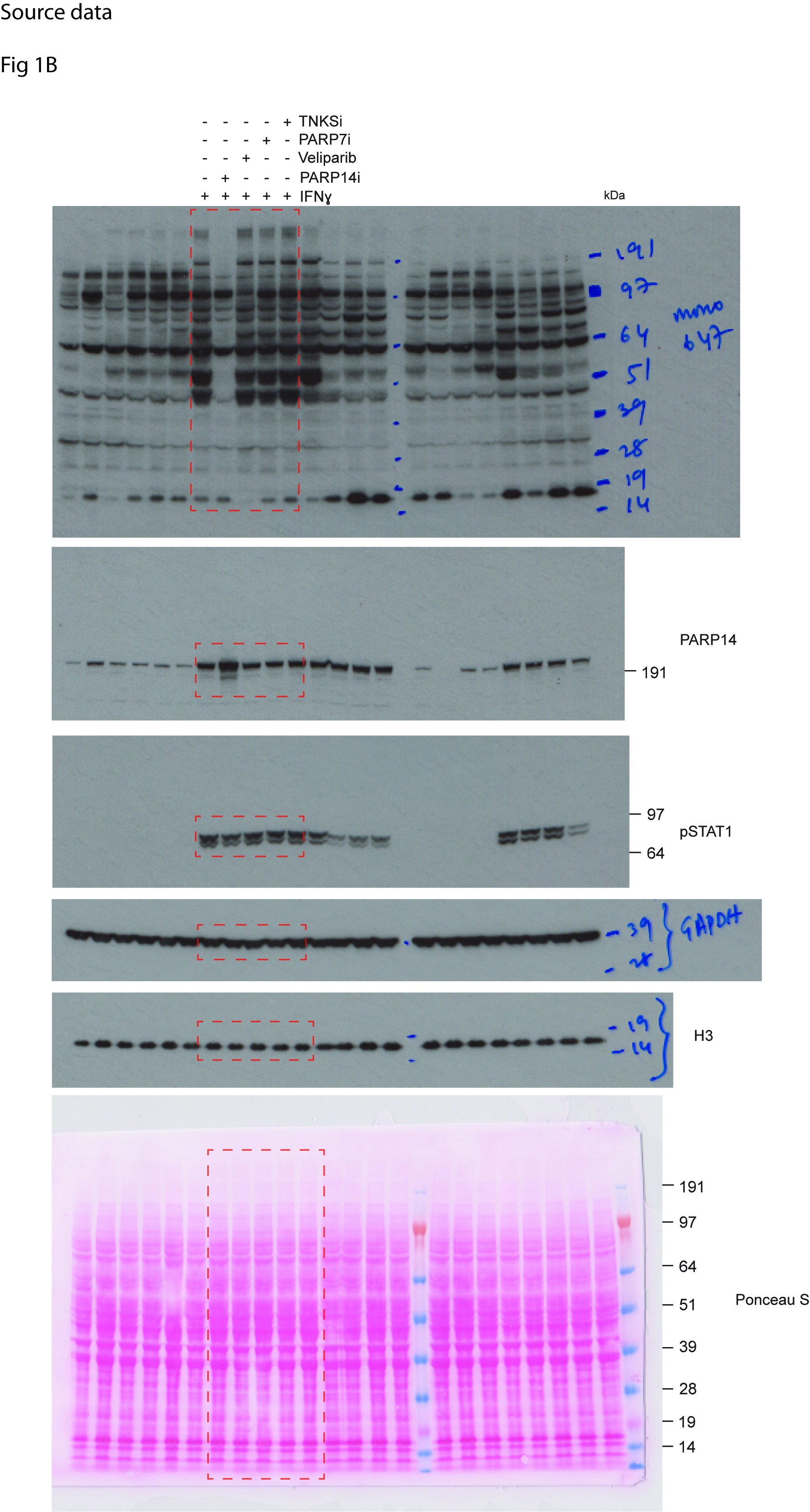

Supplement: Supplementary file 3 — Source data Fig. 1 [file 44318_2024_126_MOESM3_ESM.zip › Figure 1/FIG 1B.tif]

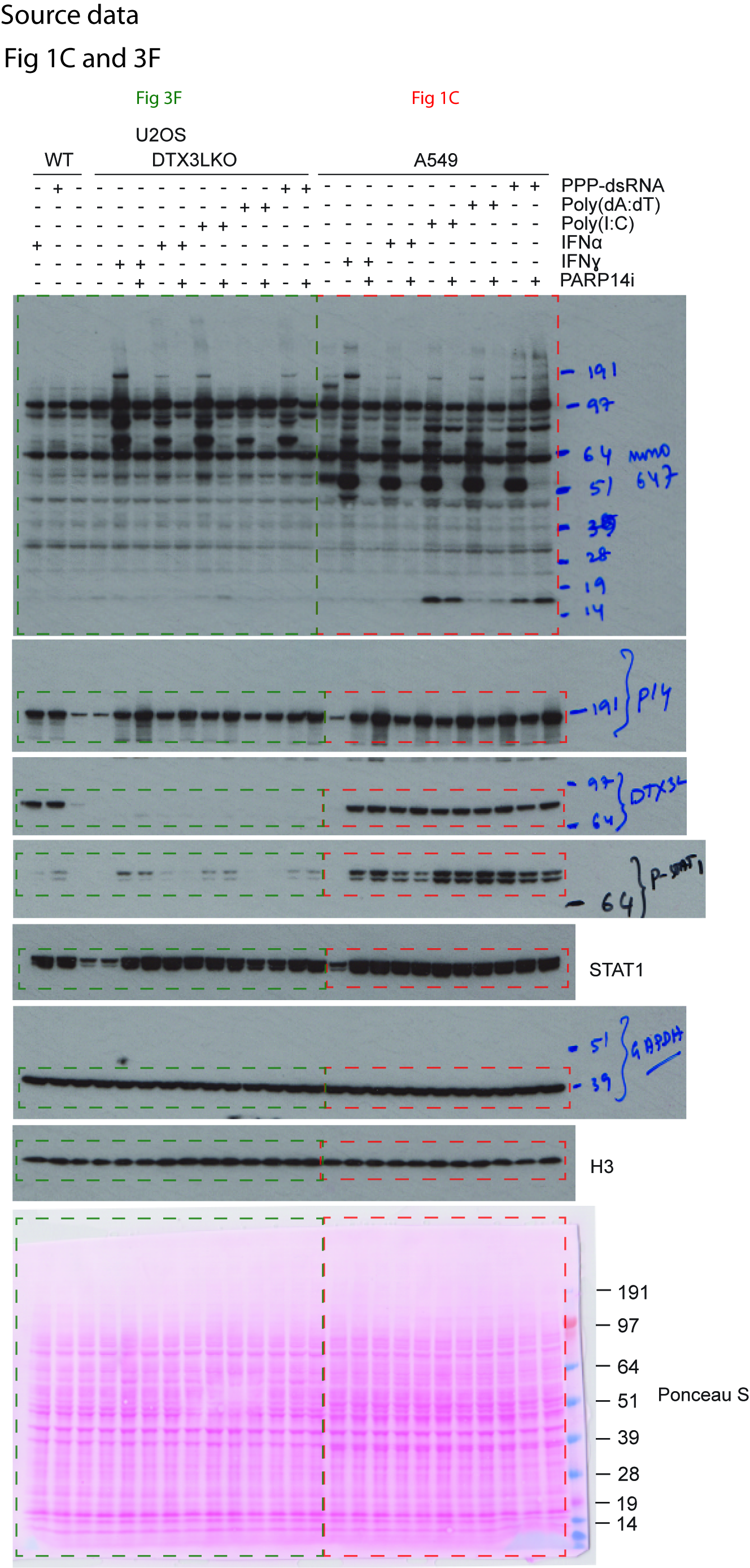

Supplement: Supplementary file 3 — Source data Fig. 1 [file 44318_2024_126_MOESM3_ESM.zip › Figure 1/Fig 1C.tif]

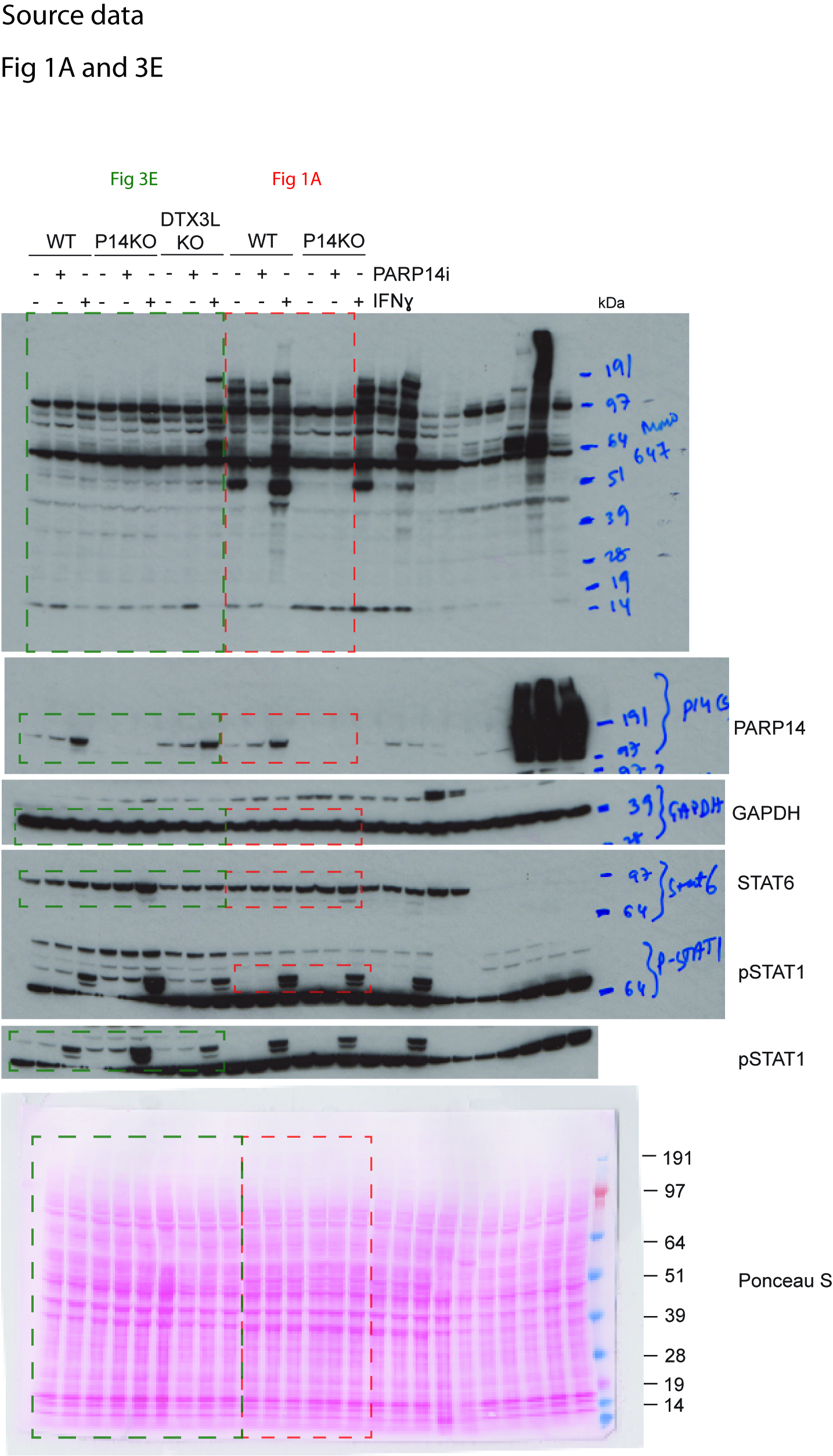

Supplement: Supplementary file 3 — Source data Fig. 1 [file 44318_2024_126_MOESM3_ESM.zip › Figure 1/Fig 1A.tif]

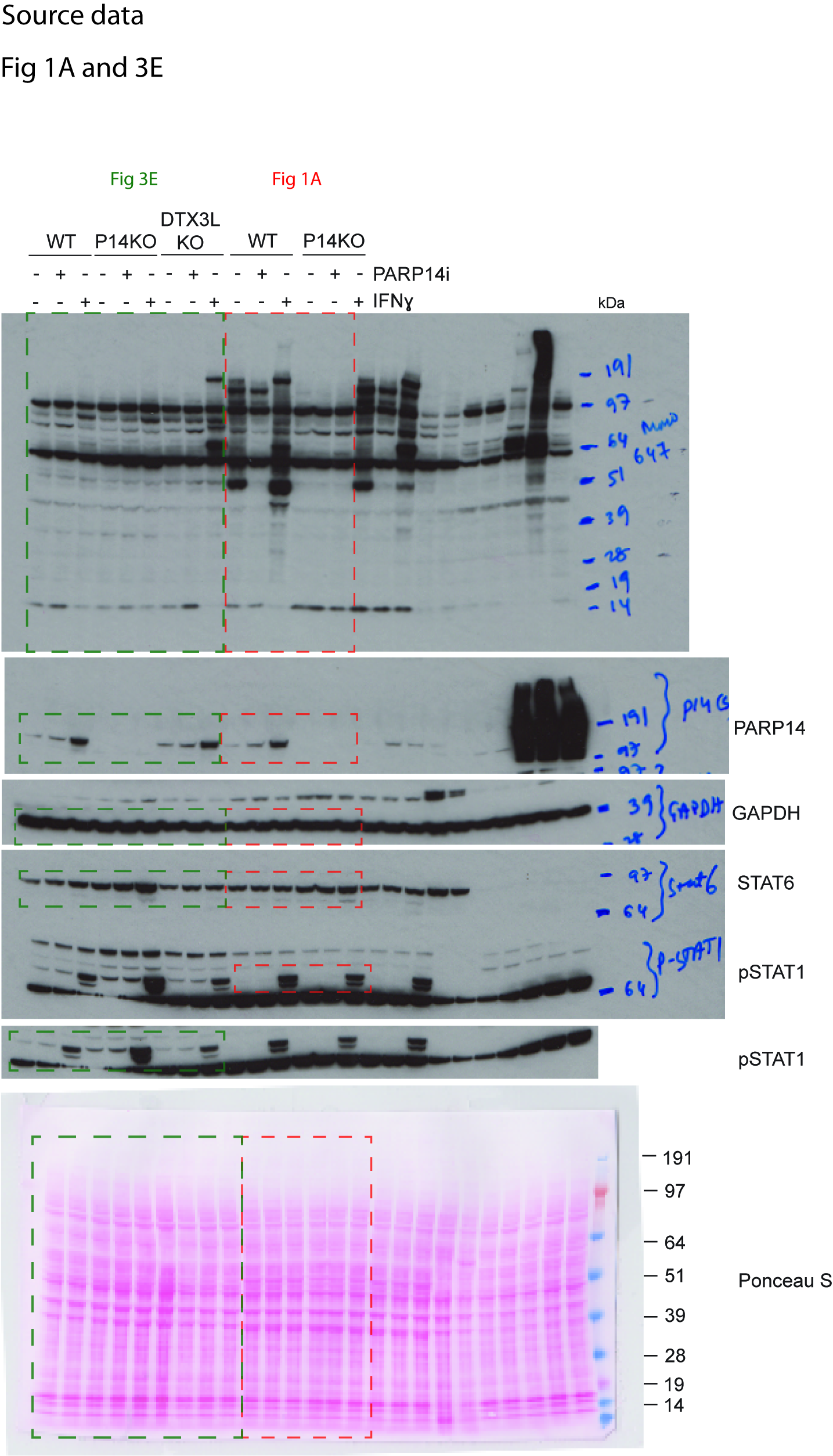

Supplement: Supplementary file 5 — Source data Fig. 3 [file 44318_2024_126_MOESM5_ESM.zip › Figure 3/Fig 3E.tif]

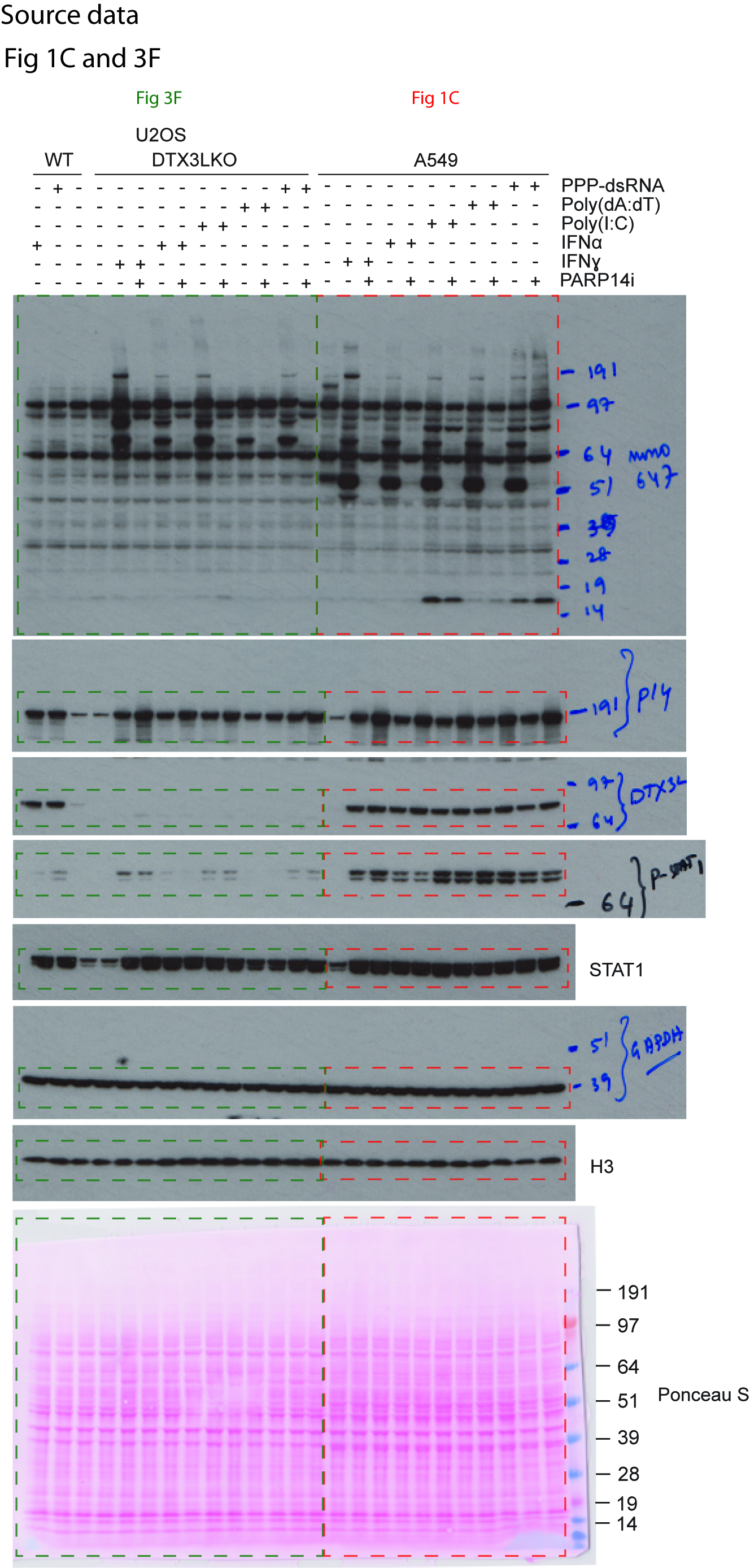

Supplement: Supplementary file 5 — Source data Fig. 3 [file 44318_2024_126_MOESM5_ESM.zip › Figure 3/Fig 3F.tif]

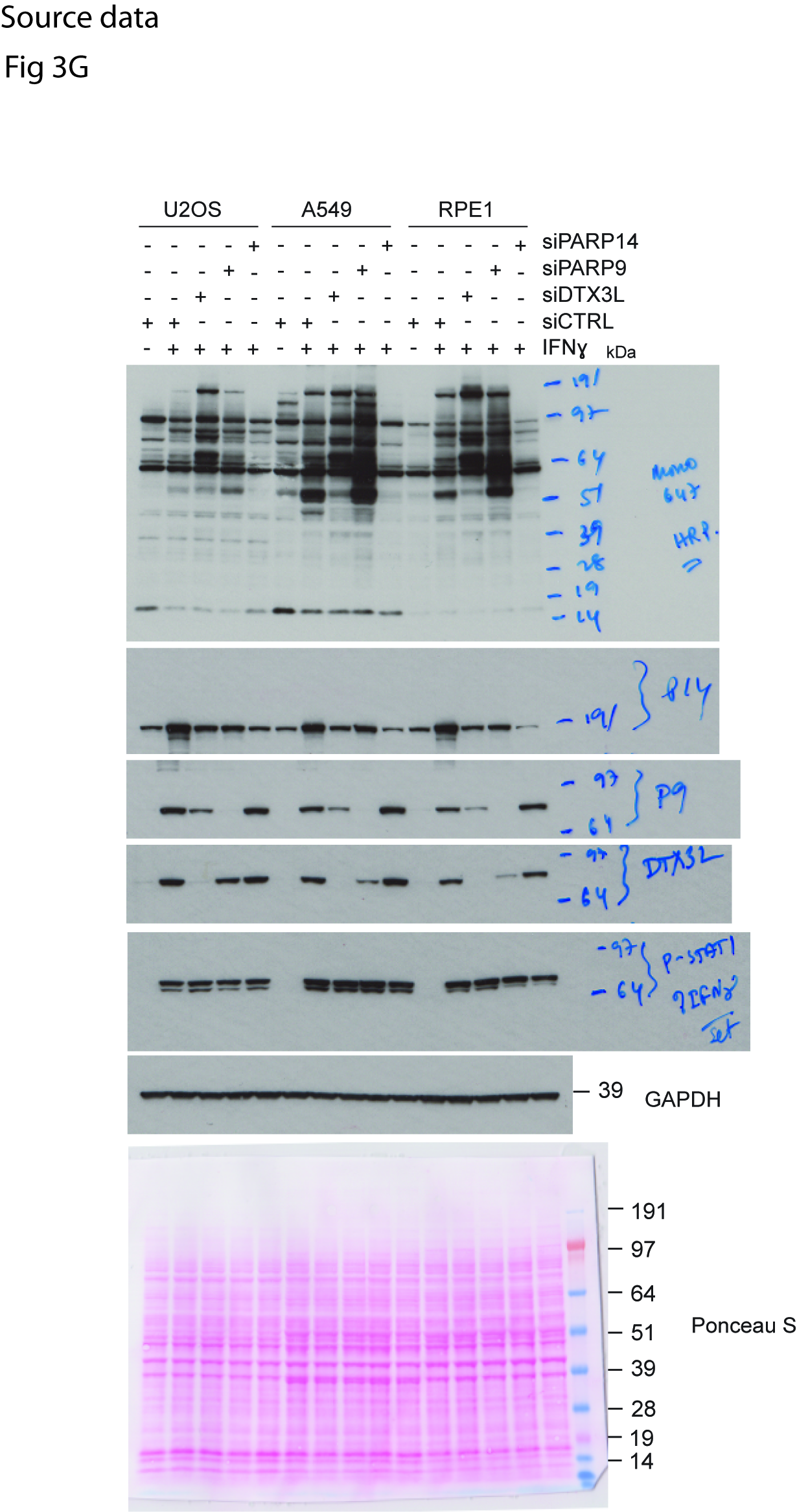

Supplement: Supplementary file 5 — Source data Fig. 3 [file 44318_2024_126_MOESM5_ESM.zip › Figure 3/FIG 3G.tif]

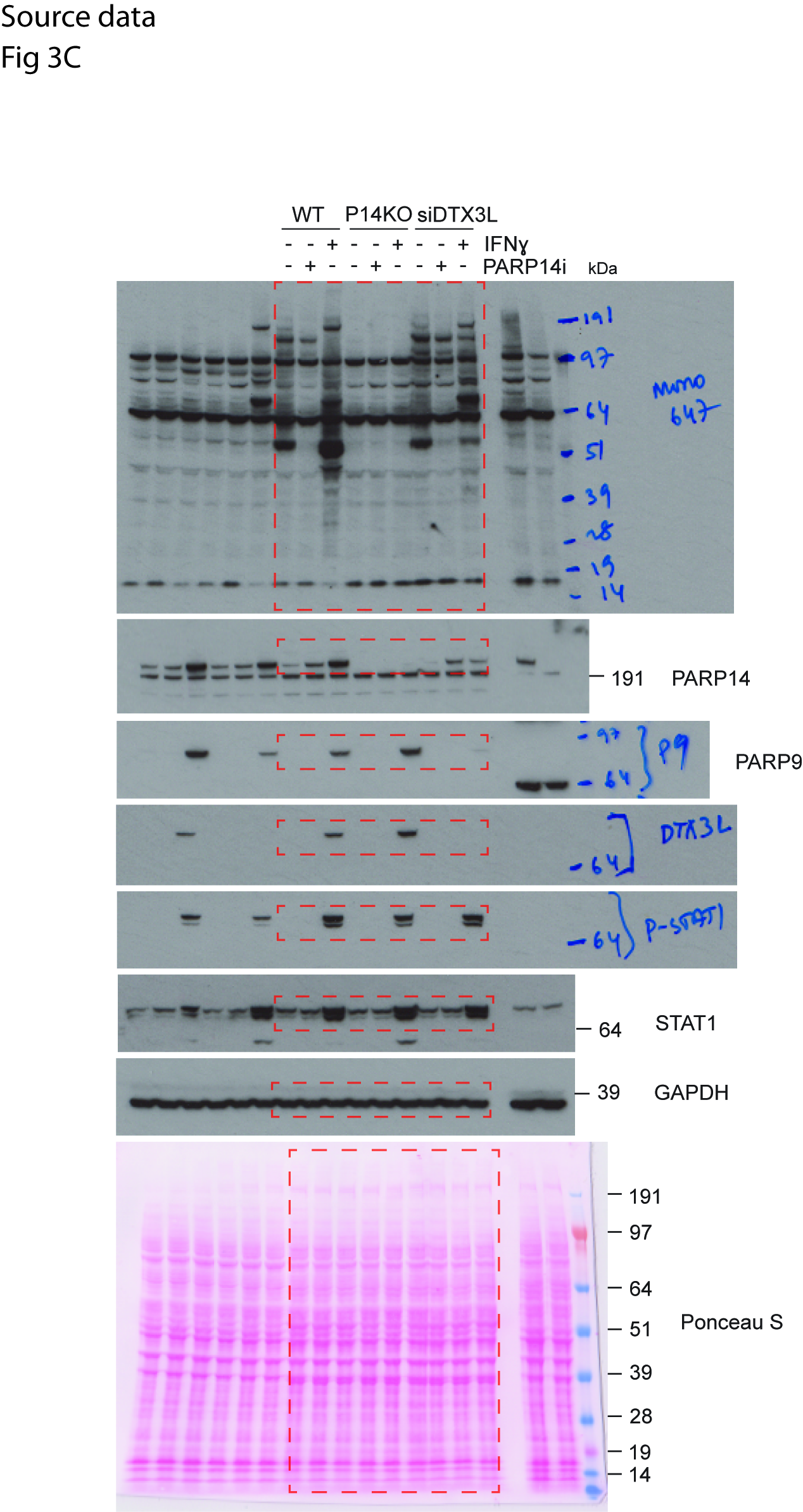

Supplement: Supplementary file 5 — Source data Fig. 3 [file 44318_2024_126_MOESM5_ESM.zip › Figure 3/3C.tif]

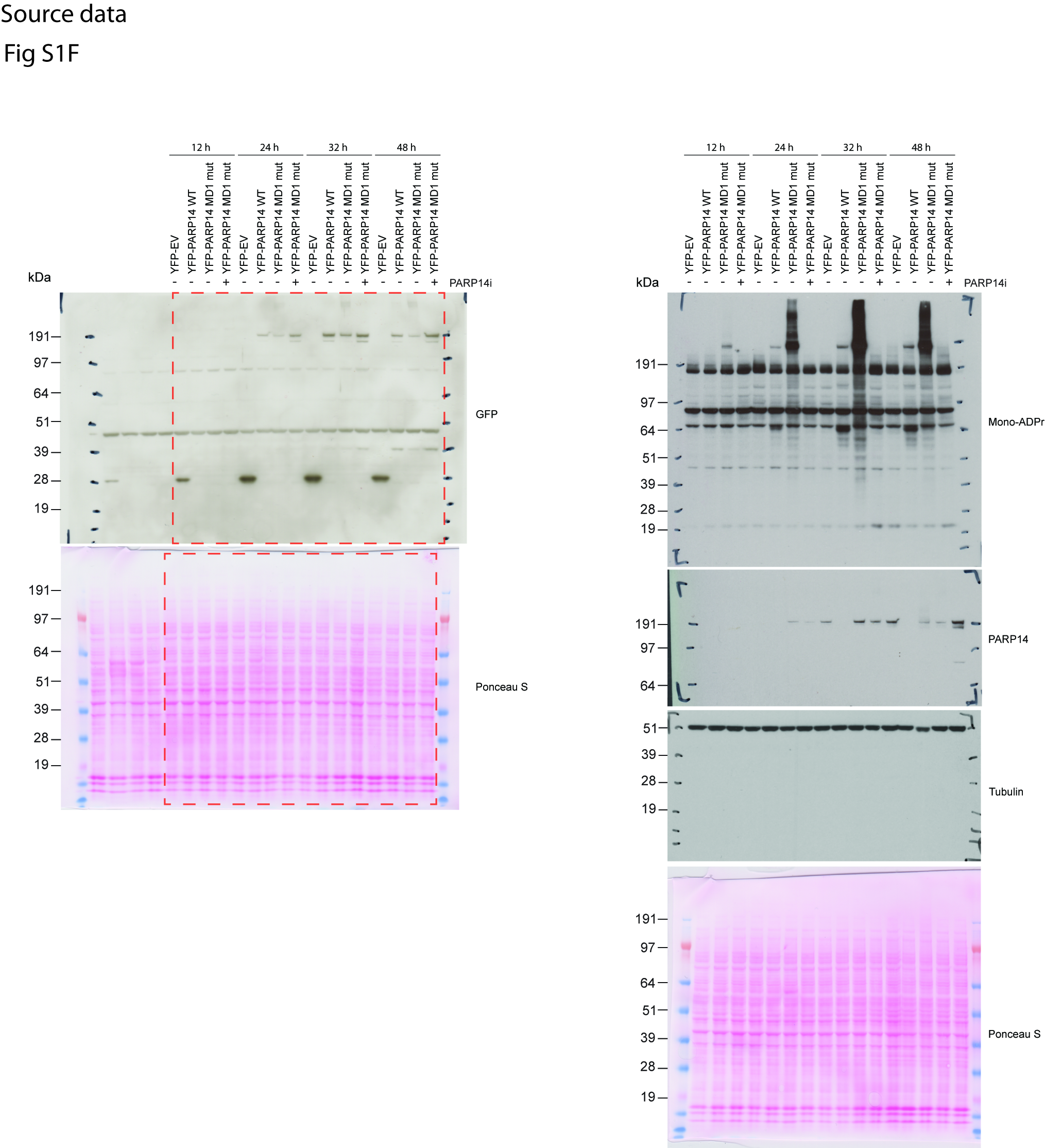

Supplement: Supplementary file 7 — Figure EV1-4 source data [file 44318_2024_126_MOESM7_ESM.zip › EV 1/EV 1F.tif]

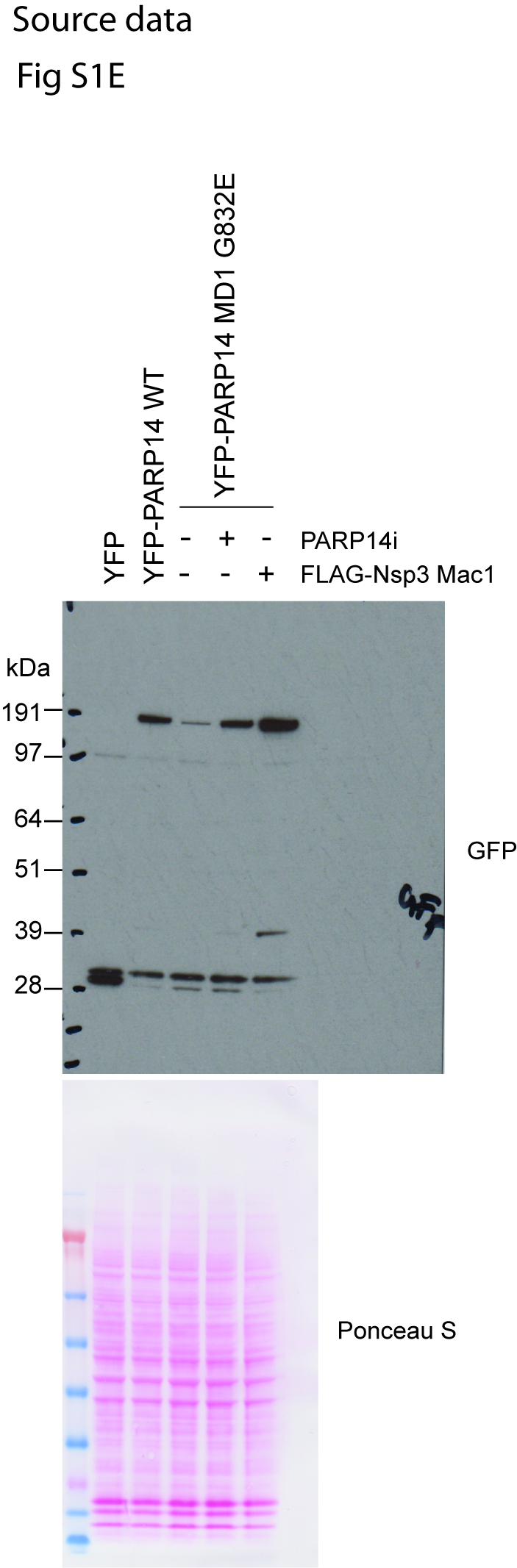

Supplement: Supplementary file 7 — Figure EV1-4 source data [file 44318_2024_126_MOESM7_ESM.zip › EV 1/EV 1E.tif]

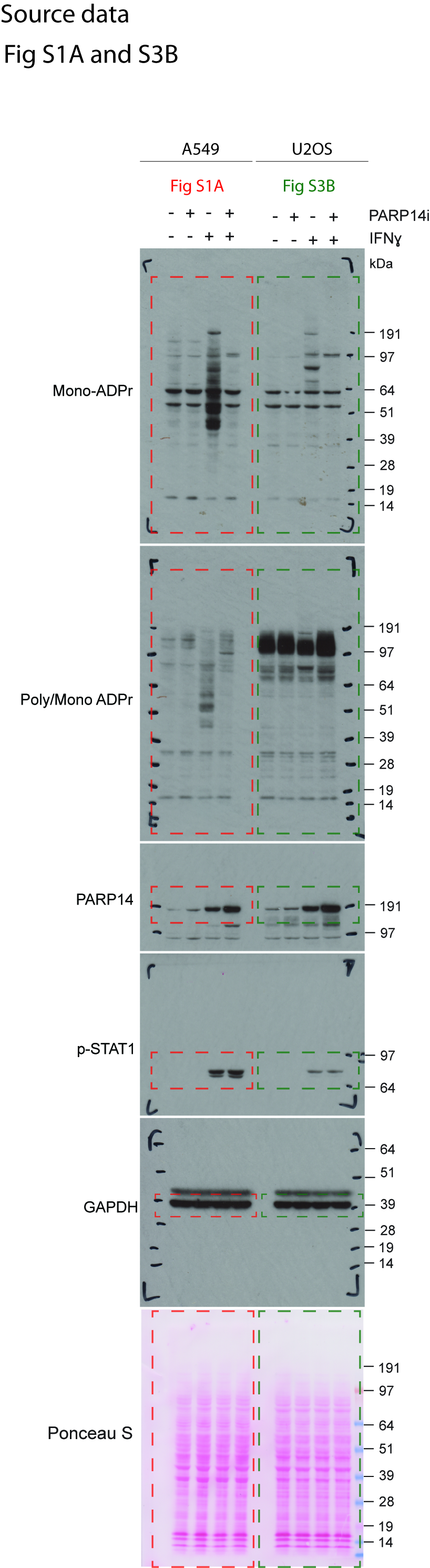

Supplement: Supplementary file 7 — Figure EV1-4 source data [file 44318_2024_126_MOESM7_ESM.zip › EV 1/EV 1A.tif]

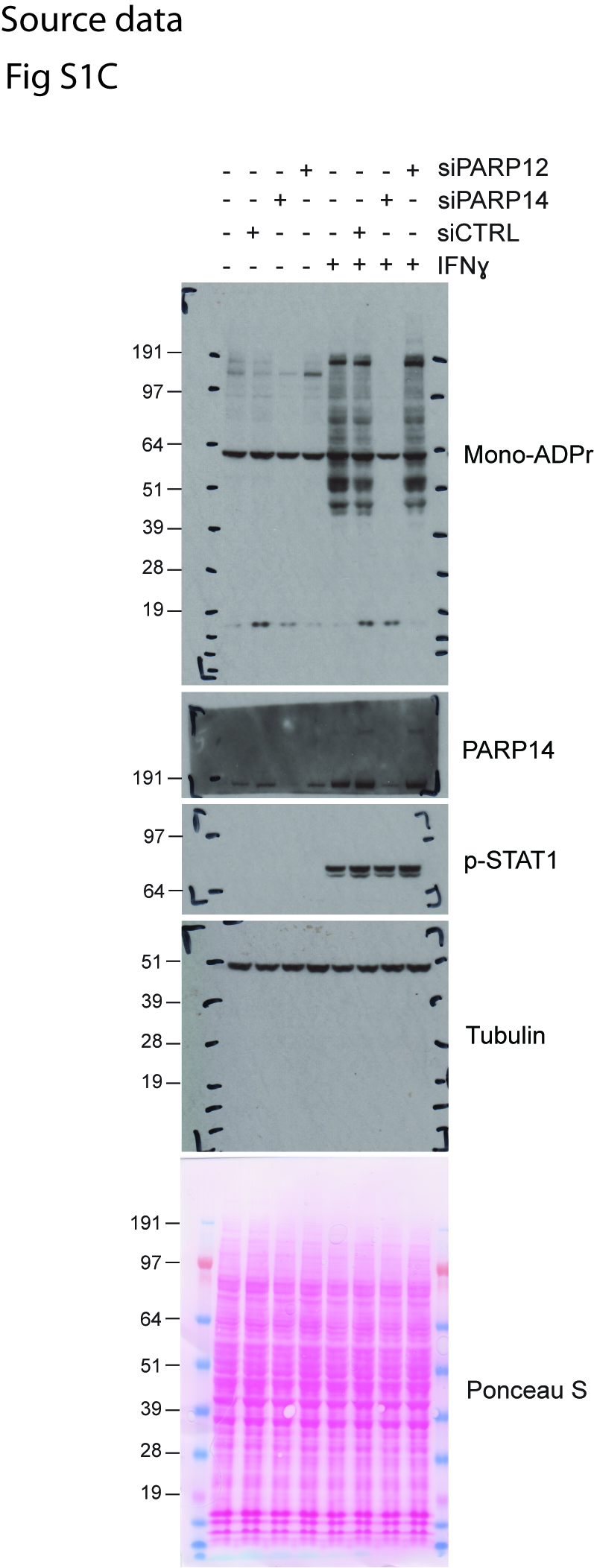

Supplement: Supplementary file 7 — Figure EV1-4 source data [file 44318_2024_126_MOESM7_ESM.zip › EV 1/EV 1C.tif]

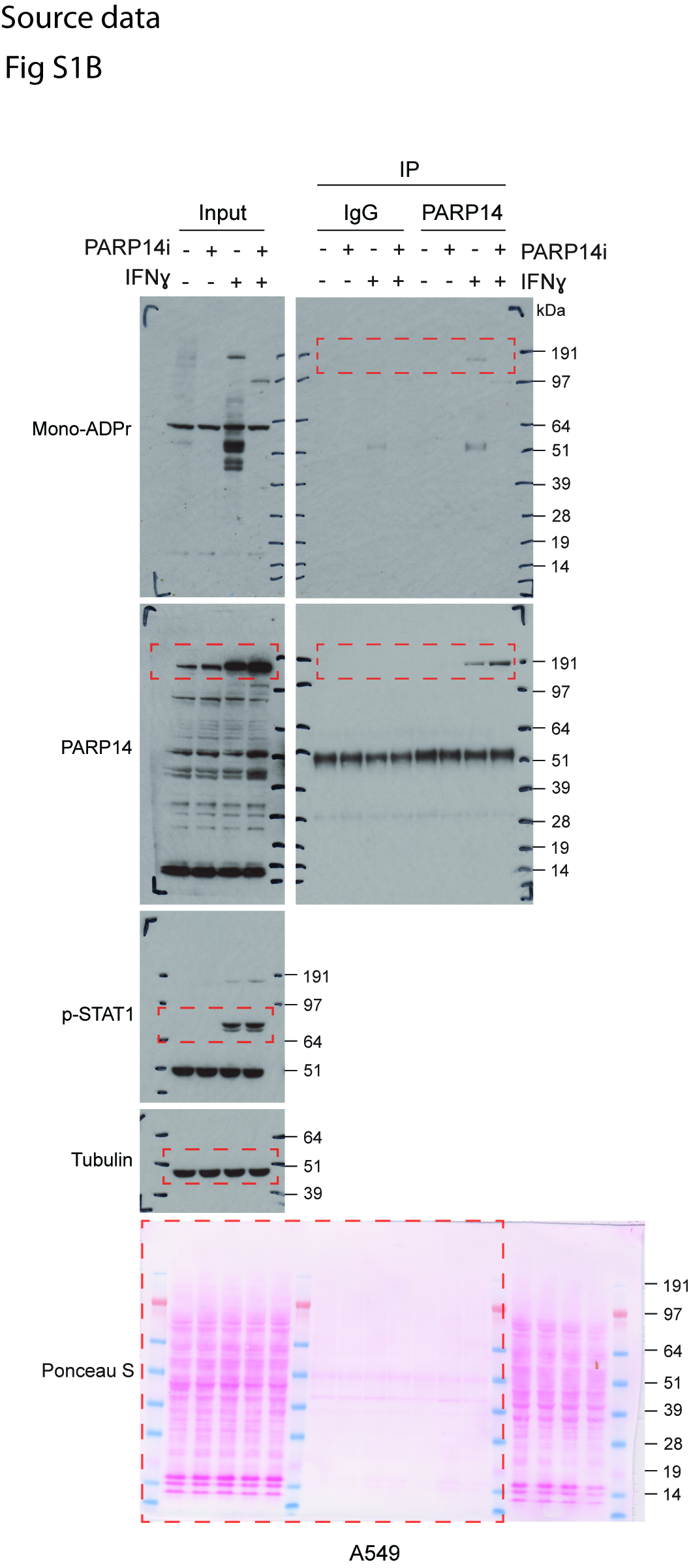

Supplement: Supplementary file 7 — Figure EV1-4 source data [file 44318_2024_126_MOESM7_ESM.zip › EV 1/EV 1B.tif]

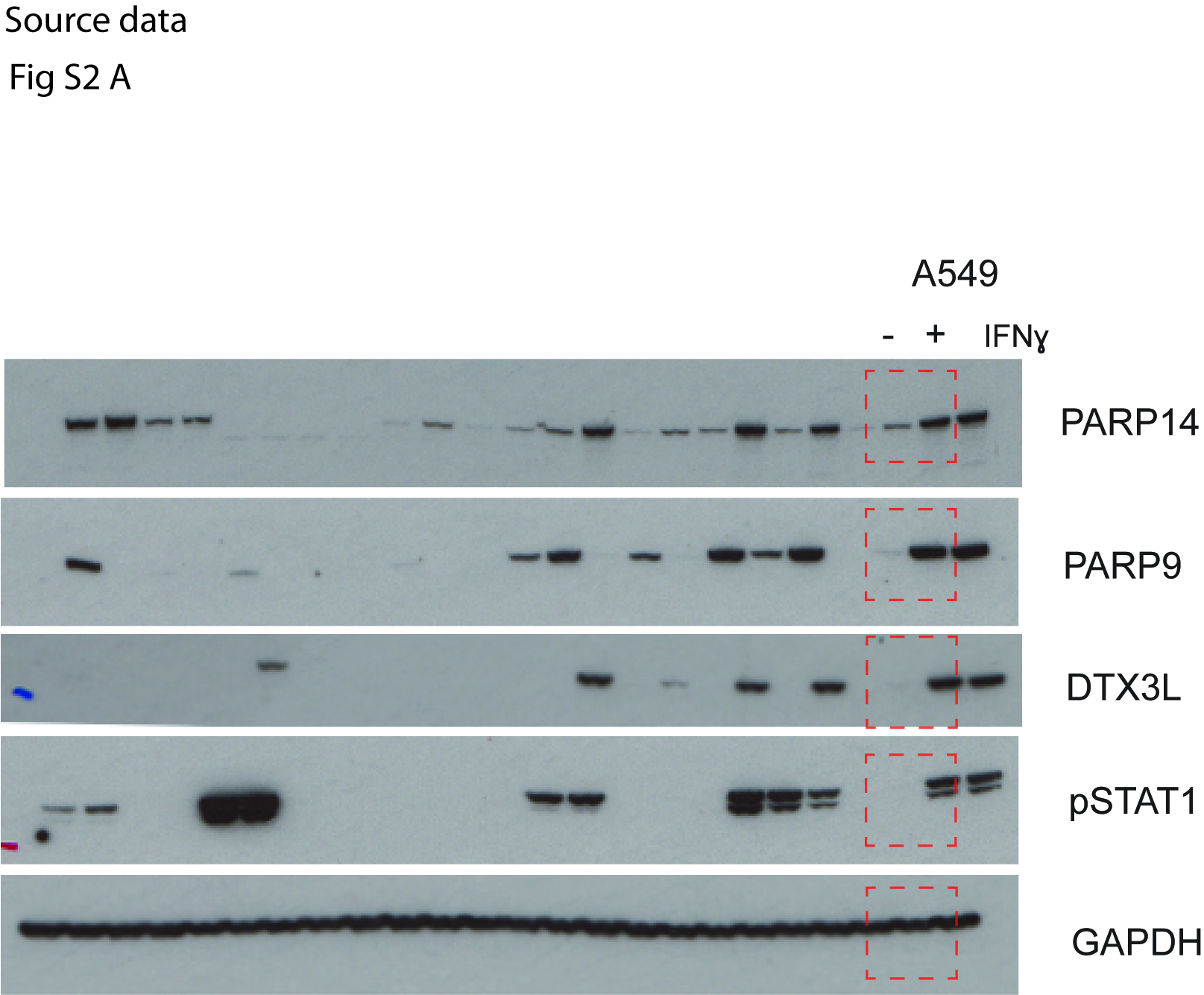

Supplement: Supplementary file 7 — Figure EV1-4 source data [file 44318_2024_126_MOESM7_ESM.zip › EV 2/EV 2A.tif]

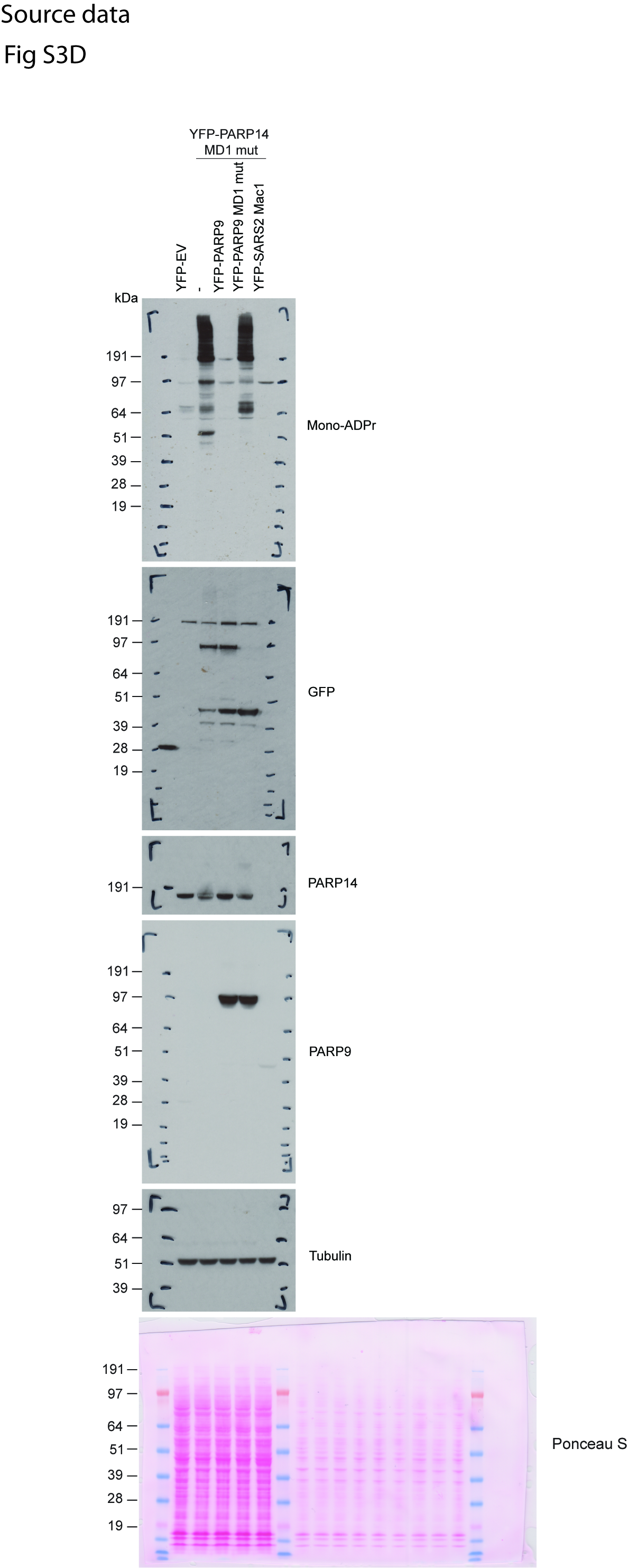

Supplement: Supplementary file 7 — Figure EV1-4 source data [file 44318_2024_126_MOESM7_ESM.zip › EV 3/EV 3D.tif]

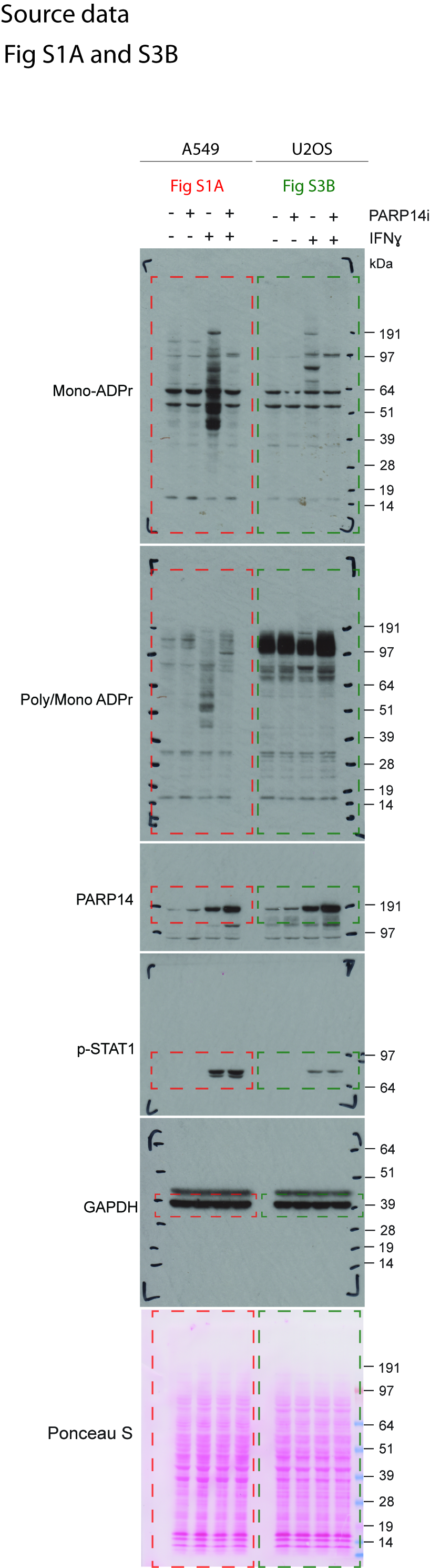

Supplement: Supplementary file 7 — Figure EV1-4 source data [file 44318_2024_126_MOESM7_ESM.zip › EV 3/EV 3B.tif]

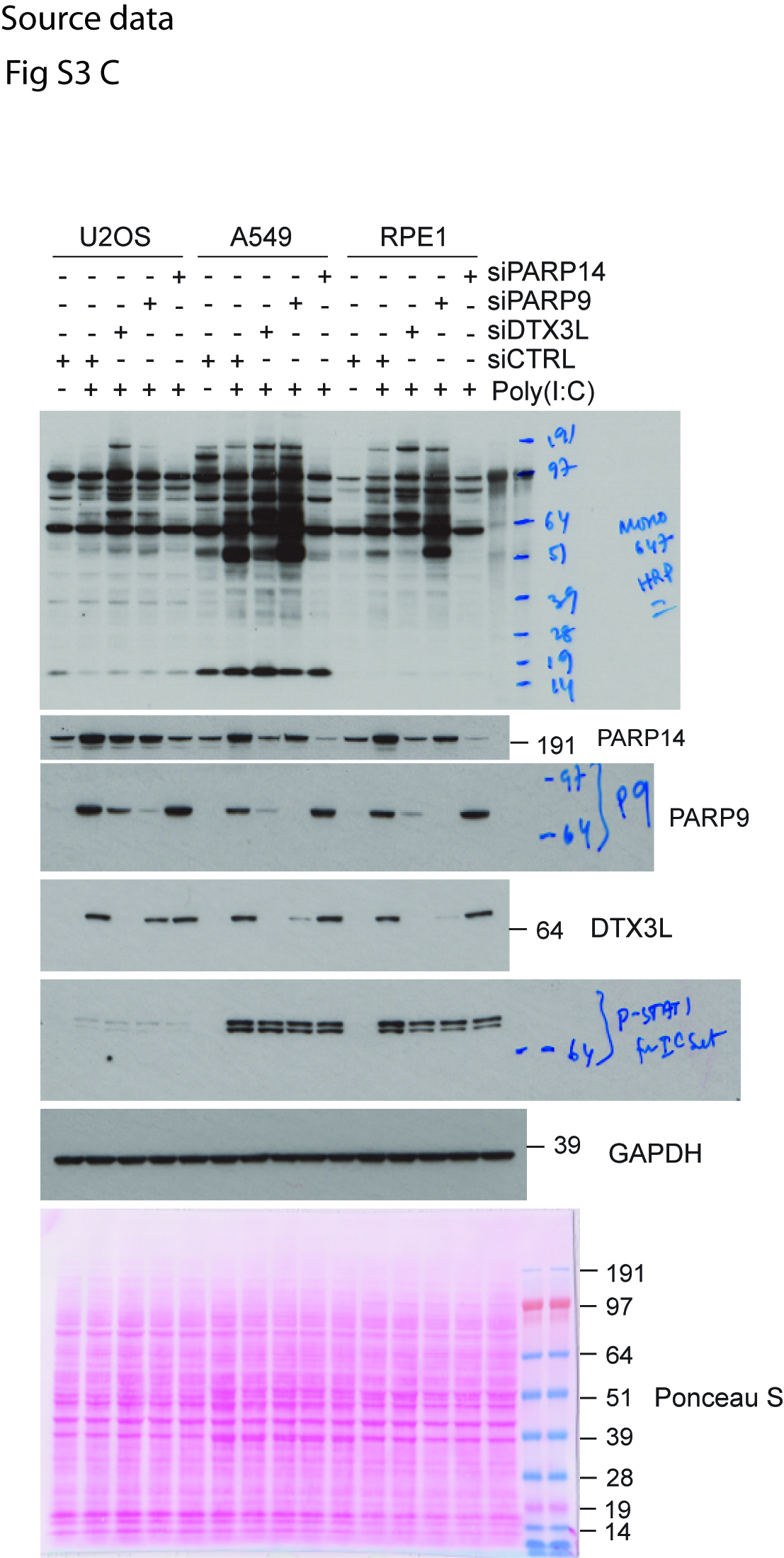

Supplement: Supplementary file 7 — Figure EV1-4 source data [file 44318_2024_126_MOESM7_ESM.zip › EV 3/EV 3C.tif]
