# Supplementary material for: PARP14 and PARP9/DTX3L regulate interferon-induced ADP-ribosylation
Source: EMBO J. 2024 Jun 4;43(14):7. doi: 10.1038/s44318-024-00126-0 (PMC11251020; doi:10.1038/s44318-024-00126-0)
Supplement: Supplementary file 8 — Expanded View Figures [file 44318_2024_126_MOESM8_ESM.pdf]

Expanded View Figures

Figure EV1. PARP14 is auto-ADPr upon immune stimulation and this regulates its stability.

(**A**) A549 cells were treated with 0.5 µM PARP14i (RBN012759) and/or with IFNɣ (100 ng/mL). Cell lysates were examined by western blot with the indicated antibodies. (**B**) A549 cells were treated with 0.5 µM PARP14i (RBN012759) and/or with IFNɣ (100 ng/mL). Cell lysates were subjected to immunoprecipitation with Protein G beads conjugated with indicated antibodies. The ADPr signal was analysed using western blotting. (**C**) A549 cells depleted or not of PARP14 or PARP12 were treated with IFNɣ (100 ng/mL) where indicated. Cell lysates were examined by western blot with the indicated antibodies. (**D**) Relative gene expression of *PARP12* for (**C**) in unstimulated and IFNɣ (100 ng/mL) stimulated A549 cells was determined by RT-qPCR. Gene expression levels were normalized to the expression of *GAPDH*. Error bars indicate average S.D. from three independent replicates. Asterisks indicate statistical significance compared with the control, as determined by Welch's t-test (ns: not significant, **$p < 0.01$, ***$p < 0.001$ Two-tailed P value, siCTRL vs siPARP12 -IFNɣ $p = 0.002$, siCTRL vs siPARP12 +IFNɣ $p = 0.0006$). (**E**) U2OS cells were transfected with the indicated plasmids in the presence or absence of 0.5 µM PARP14i or expression of FLAG-SARS2 Mac1. The levels of YFP-tagged PARP14 in cell lysates were assessed using an anti-GFP antibody. (**F**) U2OS cells were transfected with indicated plasmids in the presence or absence of PARP14 inhibitor (0.5 µM). Cells were lysed 12, 24, 32 or 48 h after transfection and analysed by western blot using the indicated antibodies. For all blots, pSTAT1 antibody was used as a positive control for stimulation of immune response. GAPDH or tubulin were used as a loading control. Source data are available online for this figure.

© The Author(s)

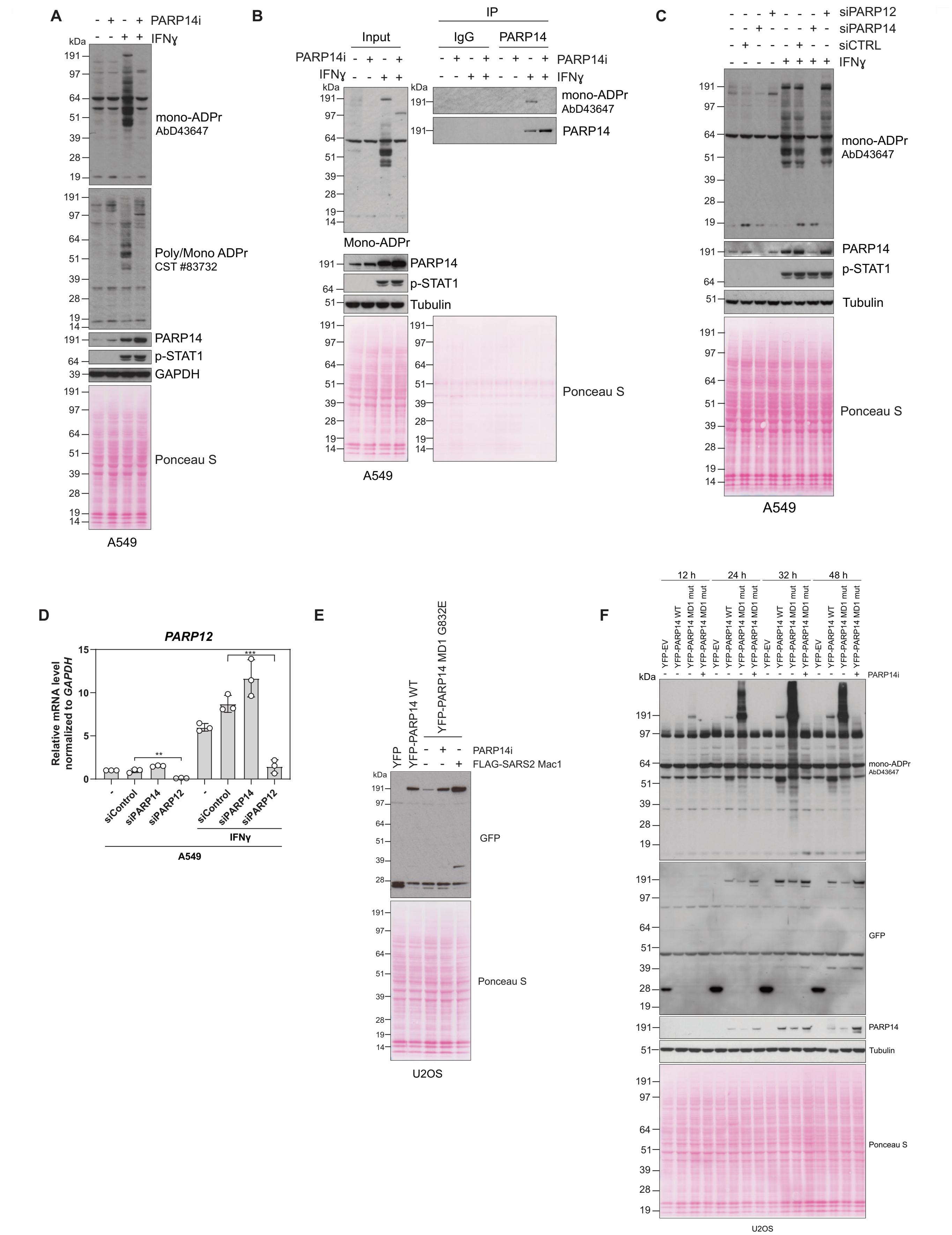
A
PARP14i
IFNɣ
kDa
mono-ADPr
AbD43647
Poly/Mono ADPr
CST #83732
PARP14
p-STAT1
GAPDH
Ponceau S
A549
B
IP
Input
IgG
PARP14
PARP14i
IFNɣ
mono-ADPr
AbD43647
PARP14
Mono-ADPr
PARP14
p-STAT1
Tubulin
Ponceau S
A549
C
siPARP12
siPARP14
siCTRL
IFNɣ
mono-ADPr
AbD43647
PARP14
p-STAT1
Tubulin
Ponceau S
A549
D
PARP12
Relative mRNA level normalized to GAPDH
siControl
siPARP14
siPARP12
IFNγ
A549
E
YFP
YFP-PARP14 WT
YFP-PARP14 MD1 G832E
PARP14i
FLAG-SARS2 Mac1
GFP
Ponceau S
U2OS
F
12 h
24 h
32 h
48 h
YFP-EV
YFP-PARP14 WT
YFP-PARP14 MD1 mut
PARP14i
mono-ADPr
AbD43647
GFP
PARP14
Tubulin
Ponceau S
U2OS

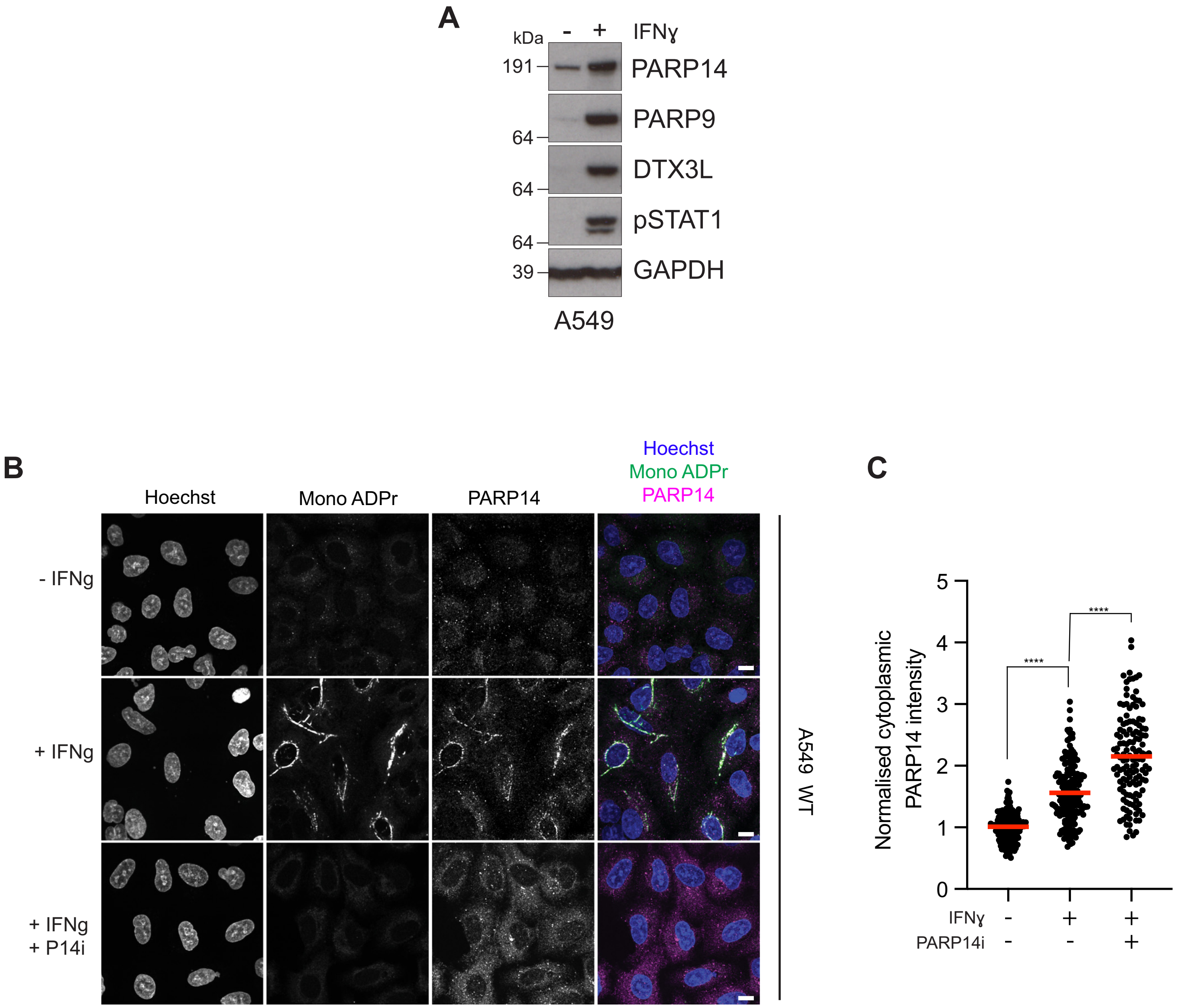

Figure EV2. PARP14, PARP9 and DTX3L increase after IFNɣ stimulation.

(**A**) A549 cells were treated or not with IFNɣ (100 ng/mL). Cell lysates were analysed by western blot using the indicated antibodies. GAPDH was used as a loading control. pSTAT1 indicates induction of the interferon response. (**B**) Confocal images showing A549 cells untreated or treated with IFNɣ (100 ng/mL) in the presence or absence of PARP14i (0.5 µM). Cells were stained with Hoechst (Blue), mono-ADPr (AbD43647 IgG-coupled) (Green) and PARP14 (Abcam, ab224352) (Magenta). Scale bars, 20 µm. (**C**) Normalised intensity of cytoplasmic PARP14 signal from (**B**). Fluorescence intensity was normalised to WT cells in the absence of IFNɣ or PARP14i. The red line indicates mean intensity. Statistical analysis was determined using a Kruskal Wallis test with Bonferroni correction followed by post hoc Dunn's test. Asterisks indicate statistical significance (****$p < 0.0001$). Data are representative of two independent replicates. Source data are available online for this figure.

 © The Author(s)

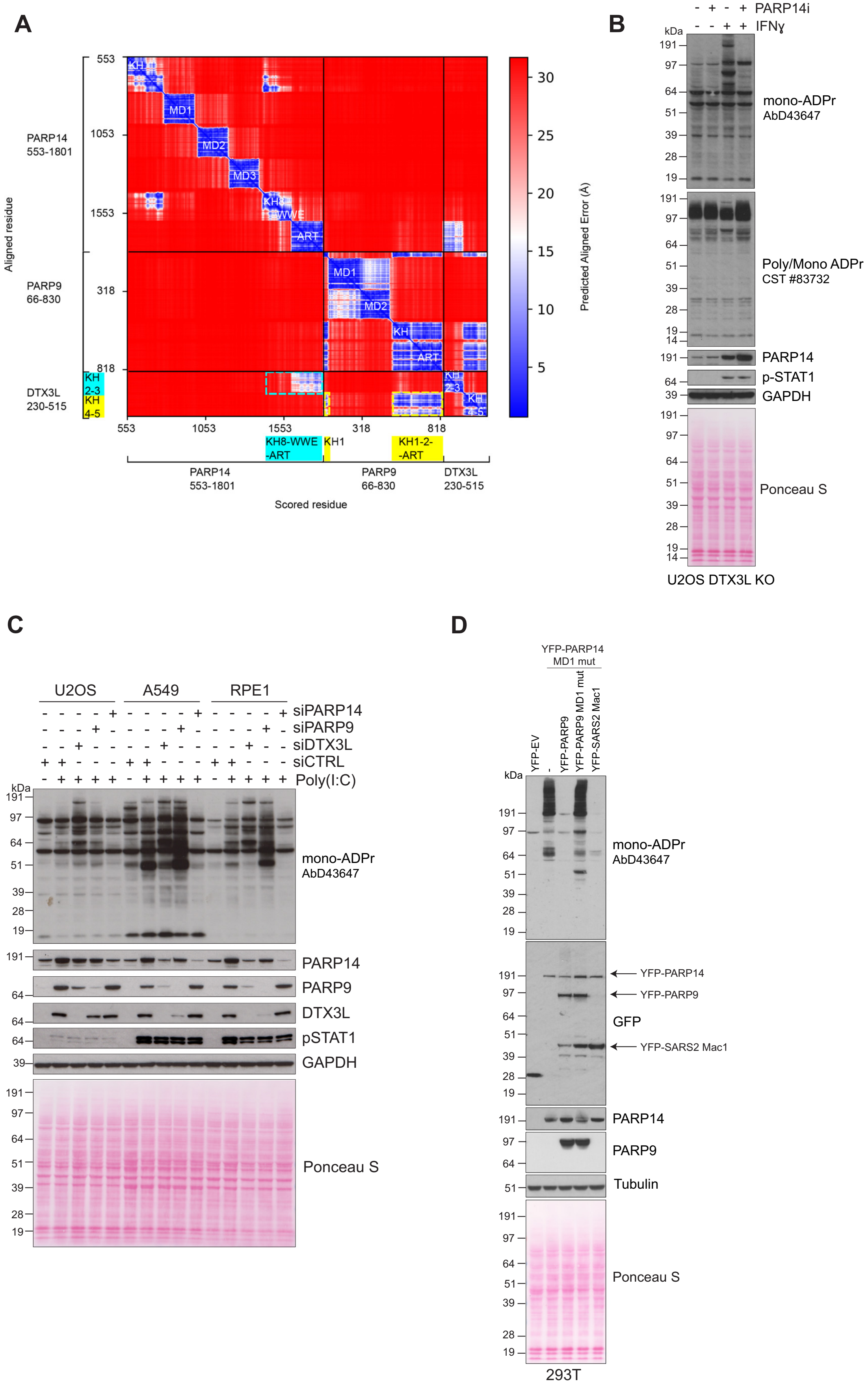
A
Aligned residue
PARP14
553-1801
PARP9
66-830
DTX3L
230-515
553
1053
1553
318
818
KH
MD1
MD2
MD3
WWE
ART
KH 2-3
KH 4-5
KH8-WWE -ART
KH1
KH1-2- -ART
Scored residue
Predicted Aligned Error (Å)
30
25
20
15
10
5
B
PARP14i
IFNɣ
kDa
mono-ADPr
AbD43647
Poly/Mono ADPr
CST #83732
PARP14
p-STAT1
GAPDH
Ponceau S
U2OS DTX3L KO
C
U2OS
A549
RPE1
siPARP14
siPARP9
siDTX3L
siCTRL
Poly(I:C)
mono-ADPr
AbD43647
PARP14
PARP9
DTX3L
pSTAT1
GAPDH
Ponceau S
D
YFP-PARP14
MD1 mut
YFP-EV
YFP-PARP9
YFP-PARP9 MD1 mut
YFP-SARS2 Mac1
mono-ADPr
AbD43647
YFP-PARP14
YFP-PARP9
YFP-SARS2 Mac1
GFP
PARP14
PARP9
Tubulin
Ponceau S
293T

© The Author(s)

Figure EV3. PARP9/DTX3L regulate PARP14-dependent ADPr.

(**A**) Predicted aligned error (PAE) plot of PARP14 (553–1801)/DTX3L (230–515)/PARP9 (66–830) model. Inter-domain interaction between PARP14 KH8-WWE-ART and DTX3L KH2-3 is indicated in cyan dashed box. Inter-domain interaction between PARP9 KH-ART and DTX3L KH4-5 is indicated in yellow dashed box. There are also notable intra-domain contacts within each protein: PARP14 KH-WWE, PARP9 KH-ART, PARP9 tandem macrodomains, DTX3L KH2-3, and DTX3L KH4-5. The relative orientations of other domains with high PAE score are uncertain. (**B**) U2OS DTX3L KO cells were treated with 0.5 μM PARP14i and/or 100 ng/mL IFNɣ. Cell lysates were examined by western blot with the indicated antibodies. (**C**) U2OS, A549 and RPE1 cells depleted or not of DTX3L, PARP9 or PARP14 were treated with poly(I:C). Cell lysates were examined by western blot with the indicated antibodies. For all blots, pSTAT1 antibody was used as a positive control for stimulation of immune response. (**D**) 293T cells were transfected with YFP-tagged PARP14 MD1 mutant alone or co-transfected with indicated plasmids. Cell lysates were collected 24 h after transfection and examined with the western blot using indicated antibodies. GAPDH or tubulin were used as a loading control in all blots. Source data are available online for this figure.

 © The Author(s)

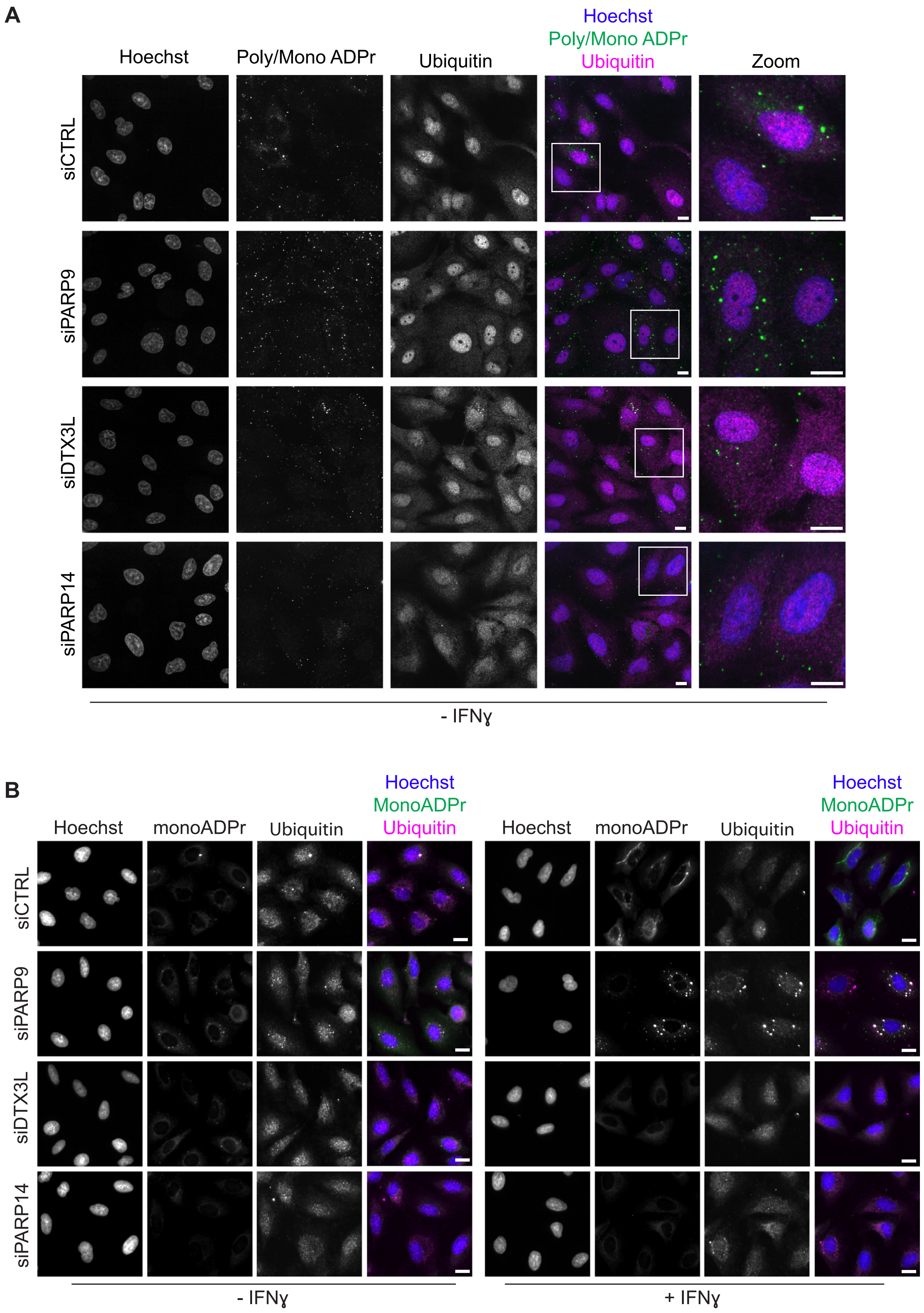
A
Hoechst
Poly/Mono ADPr
Ubiquitin
Hoechst
Poly/Mono ADPr
Ubiquitin
Zoom
siCTRL
siPARP9
siDTX3L
siPARP14
- IFNɣ
B
Hoechst
monoADPr
Ubiquitin
Hoechst
MonoADPr
Ubiquitin
Hoechst
monoADPr
Ubiquitin
Hoechst
MonoADPr
Ubiquitin
siCTRL
siPARP9
siDTX3L
siPARP14
- IFNɣ
+ IFNɣ

Figure EV4. PARP14, PARP9 and DTX3L regulate ADPr and ubiquitin foci formation.

(**A**) Confocal images showing A549 WT cells deleted of PARP9, DTX3L or PARP14 in the absence of IFNɣ. Cells were stained with Hoechst (Blue), ADPr (poly/mono-ADPr antibody, CST #83732) (Green) and ubiquitin (Magenta). (**B**) Widefield images showing A549 WT cells depleted of PARP9, DTX3L or PARP14, untreated or IFNɣ-treated (100 ng/mL). Cells were stained with Hoechst (Blue), mono-ADPr (AbD43647 IgG-coupled antibody) (Green) and ubiquitin (Abcam, ab134953) (Magenta). For all images, scale bar = 20 μm. Data information: Data are representative of a minimum of five (**A**) or three (**B**) independent replicates. Source data are available online for this figure.

 © The Author(s)

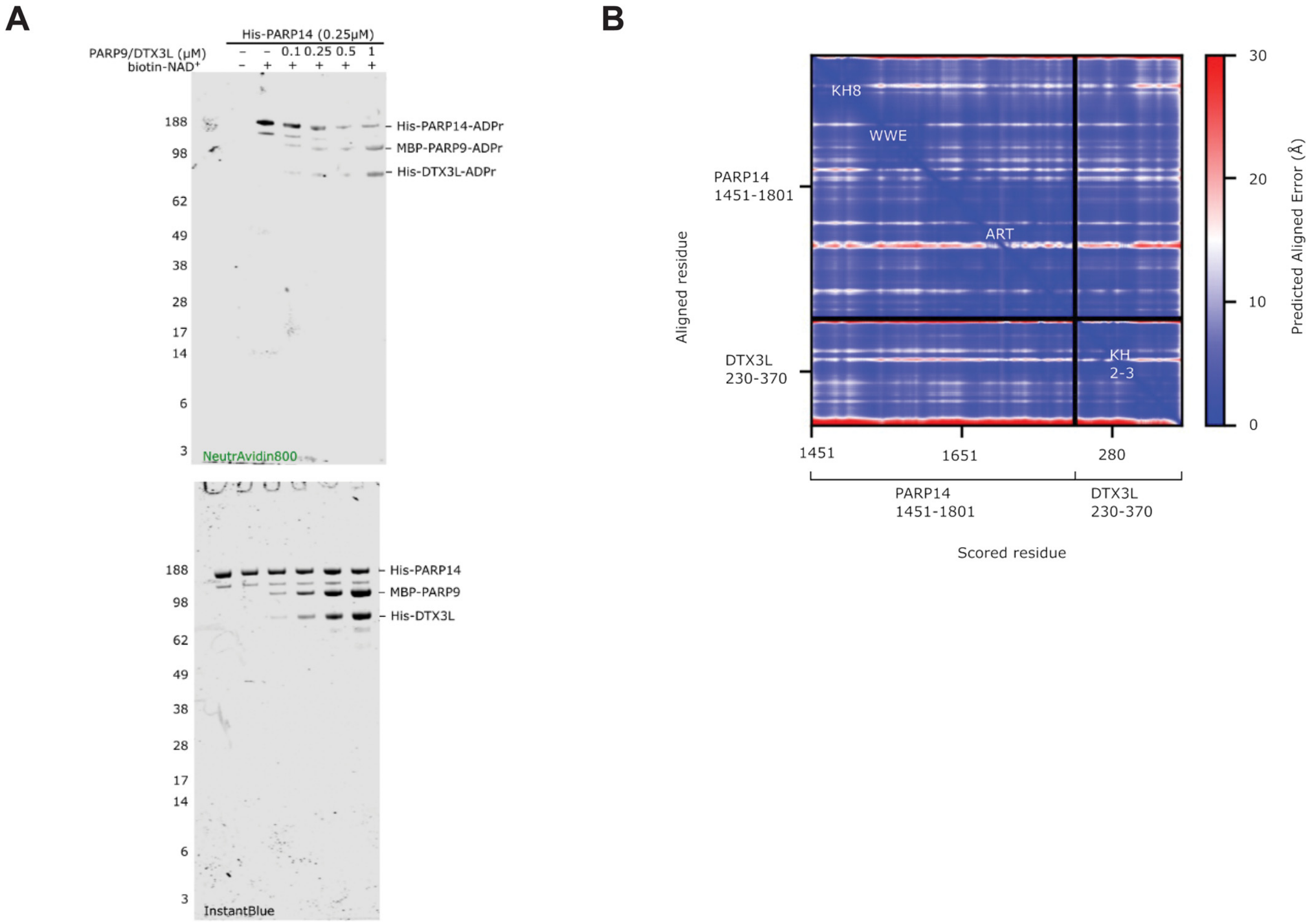

Figure EV5. DTX3L inhibits PARP14 activity in vitro.

(**A**) PARP14 FL auto-ADP-ribosylation reaction performed with biotin-NAD+ and increasing amount of PARP9/DTX3L. (**B**) Predicted aligned error plot of PARP14 KH8-WWE-ART (residue 1451–1801) and DTX3L KH2-3 (residue 230–370) model.
